# Fibrinogen‐like protein 2 in gastrointestinal stromal tumour

**DOI:** 10.1111/jcmm.17163

**Published:** 2022-01-14

**Authors:** Olli‐Pekka Pulkka, Leevi Viisanen, Olli Tynninen, Maria Laaksonen, Peter Reichardt, Annette Reichardt, Mikael Eriksson, Kirsten Sundby Hall, Eva Wardelmann, Bengt Nilsson, Harri Sihto, Heikki Joensuu

**Affiliations:** ^1^ Laboratory of Molecular Oncology Department of Oncology University of Helsinki Helsinki Finland; ^2^ Department of Pathology Helsinki University Hospital and University of Helsinki Helsinki Finland; ^3^ MediSapiens Ltd Helsinki Finland; ^4^ Sarkomzentrum Berlin‐Brandenburg, HELIOS Klinikum Berlin‐Buch Berlin Germany; ^5^ Department of Oncology Skane University Hospital and Lund University Lund Sweden; ^6^ Department of Oncology Oslo University Hospital Norwegian Radium Hospital Oslo Norway; ^7^ Gerhard‐Domagk‐Institute of Pathology University Hospital Münster Münster Germany; ^8^ Department of Surgery Sahlgrenska University Hospital Gothenburg Sweden; ^9^ Rare Cancers Research Group Department of Pathology University of Helsinki Helsinki Finland; ^10^ Comprehensive Cancer Center Helsinki University Hospital Helsinki Finland

**Keywords:** fibrinogen‐like protein 2, gastrointestinal stromal tumour, imatinib, sarcoma, tumour infiltrating lymphocytes

## Abstract

Gastrointestinal stromal tumour (GIST), the most common sarcoma of the gastrointestinal tract, can be treated effectively with tyrosine kinase inhibitors, such as imatinib. Cancer immune therapy has limited efficacy, and little is known about the immune suppressive factors in GISTs. Fibrinogen‐like protein 2 (FGL2) is expressed either as a membrane‐associated protein or as a secreted soluble protein that has immune suppressive functions. We found that GISTs expressed *FGL2* mRNA highly compared to other types of cancer in a large human cancer transcriptome database. GIST expressed FGL2 frequently also when studied using immunohistochemistry in two large clinical series, where 333 (78%) out of the 425 GISTs were FGL2 positive. The interstitial cells of Cajal, from which GISTs may originate, expressed FGL2. FGL2 expression was associated with small GIST size, low mitotic counts and low tumour‐infiltrating lymphocyte (TIL) counts. Patients whose GIST expressed FGL2 had better recurrence‐free survival than patients whose GIST lacked expression. Imatinib upregulated FGL2 in GIST cell lines, and the patients with FGL2‐negative GIST appeared to benefit most from long duration of adjuvant imatinib. We conclude that GISTs express FGL2 frequently and that FGL2 expression is associated with low TIL counts and favourable survival outcomes.

## 1 INTRODUCTION

Gastrointestinal stromal tumour (GIST) is the most common sarcoma in the gastrointestinal tract with an incidence of about 10 cases per million per year.^1^ Activating mutations in *KIT* or *PDGFRA* (*platelet*‐*derived growth factor alpha*) are considered the key drivers of the molecular pathogenesis in most GISTs, and approximately 90% of GISTs harbour an activating mutation in either *KIT* or *PDGFRA*.^2^, ^3^ Localized GISTs are usually treated with surgery, and when the risk for recurrence after surgery is estimated substantial, also with adjuvant imatinib, a tyrosine kinase inhibitor that targets a few kinases including KIT and PDGFRA.^4^ Overtly metastatic GIST is usually treated first with imatinib,^4^ but imatinib resistance often eventually emerges, and advanced GIST is usually a fatal disease.

Besides *KIT* and *PDGFRA* mutations, many other molecular aberrations likely shape the GIST phenotype. For example, small (<1 cm) GISTs (“micro‐GISTs”) that are common in the general population may harbour identical *KIT* or *PDGFRA* mutations as larger clinical GISTs, but yet have little or no malignancy potential.^5^, ^6^ Clinically detected GISTs may be associated with widely different tumour mitotic counts and survival outcomes despite identical *KIT* mutations,^3^ suggesting that the GIST phenotype is influenced by molecular factors other than the primary *KIT* or *PDGFRA* mutation. GISTs may harbour aberrations also in other genes than *KIT* or *PDGFRA*.^7^

The host immune system might also influence GIST phenotype and patient outcomes. We searched for proteins that influence the immune function and that are expressed in GIST from an in silico database that contains transcriptomes of about 20,000 human genes across 9783 human tissue samples (http://ist.medisapiens.com), and identified fibrinogen‐like protein 2 (FGL2) to be of potential interest. FGL2 is expressed in human tissues either as a membrane‐associated protein (mFGL2) that has blood coagulation activity or as a secreted soluble protein (sFGL2) that has immune‐suppressive functions regulating both the innate and the adaptive immunity.^8^, ^9^ Postnatally, constitutive FGL expression is detected commonly in T lymphocytes, and within tumours besides cancer cells also in tumour‐infiltrating leukocytes (TILs) and in the tumour microvascular endothelium.^9^ To our knowledge, the role of FGL2 has not been studied in GIST earlier. We found that FGL2 is expressed in most GISTs and in the interstitial cells of Cajal, the putative cells of origin of GISTs.^10^, ^11^ FGL2 expression was associated with low TIL counts and favourable survival outcomes. These findings suggest that FGL2 has a previously unrecognized biological role in GIST.

## 2 MATERIALS AND METHODS

### 2.1 Patients and tumour tissue samples

The human tumour tissue samples were derived from three large nonoverlapping patient cohorts. First, 598 randomly selected formalin‐fixed paraffin‐embedded tumour tissue samples consisting of 36 different histopathological cancer types were retrieved from the archives of the Department of Pathology, Helsinki University Hospital, and stained for FGL2 protein using immunohistochemistry.

The second cohort investigated was a population‐based series that consisted of 288 GIST patients diagnosed and treated in western Sweden from 1983 through 2000, and included all GIST patients diagnosed within the geographical region and time period.^12^ The associations between GIST FGL2 expression, GIST clinical and pathological features, and patient survival outcomes were investigated in this cohort. The patients underwent surgery for GIST, and none received adjuvant imatinib or other tyrosine kinase inhibitors. The median follow‐up time of the patients was 44 months after surgery. We were able to include in the current study 153 (53.1%) out of the 288 patients in the original cohort, since for the remaining 135 cases representative GIST tissue was not available, tumour histology was not compatible with GIST at histology review or we lacked the minimum clinical information required (gender, tumour diameter, tumour site in the gastrointestinal tract or patient follow‐up data). The risk stratification for GIST recurrence was done according to the U.S. National Institutes of Health consensus criteria.^13^

The third patient cohort studied consisted of GIST patients who participated in the Scandinavian Sarcoma Group (SSG) XVIII adjuvant trial^14^, ^15^ (ClinicalTrials.gov identifier, NCT00116935). The trial is an open label, multicentre, phase 3 study. The patients had KIT‐positive, operable GIST, and they were at a high risk for GIST recurrence according to the modified National Institute of Health Criteria. After surgery for GIST, 400 patients were randomly allocated to receive adjuvant imatinib 400 mg per day orally for either 12 or 36 months. The trial primary end point was recurrence‐free survival (RFS). In this trial, patients who were allocated to 36 months of imatinib had statistically significantly longer RFS and overall survival compared to those scheduled for 12 months of imatinib.^15^ We excluded from this cohort three patients who were randomized without the patient signing informed consent and 15 patients who did not have GIST at central pathology review. Of the remaining 382 patients, 272 (71.2%) had representative GIST tissue available to study FGL2 expression. The median follow‐up time of the patients alive was 10 years after the date of randomization. The trial was approved by the national or regional ethics boards or the institutional review committees of the participating study sites. An institutional review board approved the use of the tissue samples for the study (HUS 38/13/03/02/2015 and HUS 9/13/03/02/2014). The study workflow and the use of tumour tissue samples from the three patient series are depicted in Figure S1.

### 2.2 *FGL2* mRNA expression in the IST online database

Genome mRNA expression of various human cancers and normal tissues was interrogated in the IST Online *in silico* database (MediSapiens). The gene tissue index (GTI) outlier statistics^16^, ^17^ was used to rank the outlier genes that are highly expressed in GIST in comparison with the other cancers and normal tissues investigated. The GTI analysis was done by comparing mRNA expression of each gene in 77 GIST samples against all other tissues with expression data available in the database. The number of reference samples varied from 10,654 to 19,986 and the number of reference tissue types from 317 to 409 in the analyses. In addition to the GTI analysis, we compared the mRNA expression profiles of the top outlier genes also visually to mRNA expression in other tumour types to identify the potentially most important genes in GIST.

### 2.3 Immunohistochemistry

Tissue microarrays were constructed from the representative parts of the formalin‐fixed paraffin‐embedded tumour tissues using either a 0.7‐mm‐diameter or a 1‐mm‐diameter needle. Protein expression was evaluated from 5‐µm‐thick tissue microarray sections using immunohistochemistry.^17^ The antibodies and their dilutions are provided in Table S1. Primary antibody binding was detected and visualized using the BrightVision Poly‐HRP anti‐Rabbit kit or anti‐Mouse kit (Immunologic BV, Duiven, The Netherlands) and 3,3′‐diaminobenzidine (ImmPACT™ DAB, Vector Laboratories, Burlingame, CA, USA). The polyclonal rabbit FGL2 antibody used (HPA021011, Sigma, St. Louis, MO, USA) binds to the FGL2 amino acid residues 111–195 and, therefore, detects the membrane‐bound form of FGL2 (mFGL2).^9^ GIST FGL2 staining turned out to be uniform in the full GIST tissue sections evaluated, and, therefore, we categorized tumour FGL2 expression simply as either negative (absent, ‐) or positive (faint staining +, or strong staining ++). Cancer cell FGL2 expression was scored. The immunostainings were analysed blinded without knowledge of the clinical or histopathological data.

### 2.4 Automated tumour infiltrating leukocyte counting

Analysis of the tumour‐infiltrating immune cells was done in the SSGXVIII trial sample series at 40× magnification from five hot spots (the most leukocyte dense areas) on full tissue sections using an ImmunoRatio software (Tampere, Finland) that calculates the percentage of positively stained cells out of the total cell count. The software identifies KIT‐positive tumour regions (the GIST cell‐containing regions), and then calculates the numbers of positively stained leukocytes within these regions. The tissue slides were first stained with an anti‐KIT antibody followed by a secondary CY2‐conjugated rabbit antibody directed to the selected leukocyte antigen (Rockland). The antibodies used are provided in Table S2. The stained slides were imaged with automated scanning using an Olympus BX50 microscope (Olympus) integrated SlideStrider objective slide scanner (JILab Inc) that registers both fluorescent and nonfluorescent light at 20× magnification.

### 2.5 GIST cell lines

GIST882 and GIST48 cell lines were kindly provided by Dr. Jonathan Fletcher (Harvard Medical School), and the GIST‐T1 cell line was purchased (Cosmo Bio). In an agreement with data from other studies, our GIST48 cell line is not fully imatinib resistant.^17^, ^18^, ^19^ Authenticity of the GIST882, GIST48 and GIST‐T1 cell lines was confirmed with DNA sequencing. The GIST cells were cultured in a humidified 5% CO_2_ atmosphere at 37°C, GIST882 and GIST48 cells in the RPMI 1640 medium (GIBCO) supplemented with 20% fetal bovine serum with 2% penicillin/streptomycin (GIBCO), and GIST‐T1 cells in a DMEM medium (Lonza) supplemented with 10% fetal bovine serum with 2% penicillin/streptomycin (GIBCO).

To establish an imatinib‐resistant version of GIST‐T1 cell line, the GIST‐T1‐IRO cell line, imatinib was first administered to the DMEM medium at a concentration of 20 nM during the logarithmic phase of the GIST‐T1 cell growth. The imatinib‐containing medium was applied onto the cells, followed by culture for 48 h, after which the medium was replaced with drug‐free DMEM medium. The administration of imatinib was continued until the cells started to grow normally at the 20 nM imatinib concentration. Thereafter, 20 to 100 nM higher imatinib concentrations were stepwise administered until the cells eventually grew at imatinib concentration of 2 µM suggesting imatinib resistance (Figure S2).

### 2.6 *FGL2* mRNA expression

RNA was extracted from the GIST cell lines with standard methods. Complementary DNA (cDNA) was synthesized using a SuperScript^®^ VILO™ cDNA Synthesis Kit (Invitrogen) according to the manufacturer's instructions. *FGL2* mRNA expression was quantified with real‐time qPCR using hydrolysis probes (hybridization probes labelled with a reporter dye and a quenching dye) in a LightCycler II 480 instrument (Roche Diagnostics GmbH). *FGL2* expression was normalized with *TBP* expression (encodes a TATA‐binding protein) as a control. cDNA was amplified in a 20‐μL PCR mixture using LightCycler 480 Probes Master reagents (Roche Diagnostics GmbH) and fluorescein‐labelled locked nucleic acid hydrolysis probe 65 or the LightCycler^®^ Yellow 555‐labeled locked nucleic acid hydrolysis probe *TBP* from a Universal ProbeLibrary Set (Roche Diagnostics GmbH). The PCR mixture contained 1× PCR buffer, 100 nmol/L of probe, and 200 nmol/L of each primer specific for the *FGL2*‐coding region (forward: 5′‐CCAAGCACTTTAAGCCATAAATC‐3′; reverse: 5′‐GGAATTAATTGCCCTATTAGATAACG‐3′) or for *TBP* as the reference DNA (forward: 5′‐TGAATCTTGGTTGTAAACTTGACC‐3′; reverse: 5′‐CTCATGATTACCGCAGCAAA‐3′). The ProbeFinder Assay Design Software (www.universalprobelibrary.com; Roche Diagnostics GmbH) was used for the design of the primers and the probes. The cycling parameters for *FGL2* consisted of an initial denaturation at 95°C for 10 minutes, followed by 45 cycles with denaturation at 95°C for 15 s, annealing at 60°C for 45 seconds and elongation at 72°C for 45 s. The Basic Relative Quantification method (Roche Diagnostics GmbH) was used to analyse the results.

### 2.7 Cell proliferation assay

Cell proliferation was assessed with the 3‐(4,5‐dimethylthiazol‐2‐yl)‐2,5‐diphenyltetrazolium bromide (MTT) assay (Roche Diagnostics).^17^ To assess cell sensitivity to imatinib, the cells were treated the following day after plating with increasing concentrations of imatinib (0.0001, 0.001, 0.01, 0.05, 0.1, 0.5, 1, 5 and 10 µmol/L). DMSO was used as a control, and cell proliferation was measured 72 hours after adding imatinib.

### 2.8 Invasion assay

To assess cell invasiveness, the upper chamber of a 24‐well transwell (Corning™ Falcon™ Cell Culture Inserts, 8.0 µm pore size, Fisher Scientific) was covered with 50 μl of 2.5 mg/ml matrigel containing 5 µg/ml fibronectin. The lower chamber of the transwell was filled with 600 µl of the RPMI 1640 medium (GIST882 and GIST48) supplemented with 20% fetal bovine serum or 600 µl of the DMEM medium (GIST‐T1 and GIST‐T1‐IRO) supplemented with 10% fetal bovine serum and 2% penicillin/streptomycin. For the assay, 30,000 GIST48 cells, 20,000 GIST882 cells and 10,000 GIST‐T1 or GIST‐T1‐IRO cells were plated in a medium without serum to the upper chamber of the transwell. Cells were incubated for 24 h, after which cell invasion was measured in three separate experiments by counting the cells within 10 photographed fields of the microscope (Leica CTR6000, Leica microsystems; magnification ×200). The number of invaded cells was expressed as the average number of invaded cells per one microscope field.

### 2.9 siRNA transfections

GIST cell lines were transfected using the Lipofectamine 2000 Transfection Reagent (Invitrogen). The cells were first serum starved for six hours in an Opti‐MEM Reduced Serum Medium (GIBCO). Transfections were done according to the manufacturer's instructions adding 5 pmol/L of siRNA onto GIST882, GIST‐T1 and GIST‐T1‐IRO cells, and 10 pmol/L of siRNA onto the GIST48 cells. The ON‐TARGET plus Human KIT siRNA and an ON‐TARGET plus Human *FGL2* siRNA (Thermo Scientific) were used for the transfections. The ON‐TARGET plus Non‐Targeting Pool (Thermo Scientific) was used as the negative control.

### 2.10 Western blot

Western blot was performed according to standard procedures.^17^ The primary antibodies and their dilutions used are provided in Table S3. Blot immunostains were scanned using a G:BOX Chemi XX9 imaging system (Syngene).

### 2.11 Statistical analysis

The inter‐rater agreement in immunohistochemical scoring of FGL2 between two independent raters (O.P.P. and O.T.) and the intrarater agreement was assessed by computing Cohen's kappa coefficient. Frequency tables were analysed using the χ2 test. Non‐normal distributions between groups were compared with the Mann‐Whitney U‐test. Cumulative survival was estimated with the Kaplan‐Meier method. Survival between groups was compared using the log‐rank‐test and the hazard ratios (HR) were computed using a univariable Cox proportional hazards model. Multivariable survival analyses were done with the Cox proportional hazards model. RFS was calculated from the date of GIST diagnosis (the western Sweden series) or from the date of randomization (the SSGXVIII series) to the date of GIST recurrence or death, whenever death preceded recurrence, censoring the patients alive on the date of last follow‐up. Disease‐specific survival was computed from the date of the diagnosis to death considered to result from GIST, censoring patients who died from another cause on the date of death and patients who were alive on the last date of follow‐up. Overall survival was calculated from the date of GIST diagnosis to the date of death, censoring patients who were alive on the last date of follow‐up. The interaction term between adjuvant imatinib treatment duration and GIST FGL2 expression was calculated with multivariable Cox regression. All *p* values are 2‐sided. The statistical calculations were done with the IBM SPSS Statistics package v. 24.0 (IBM).

## 3 RESULTS

### 3.1 FGL2 expression in GIST and other cancer types

**FIGURE 1:**
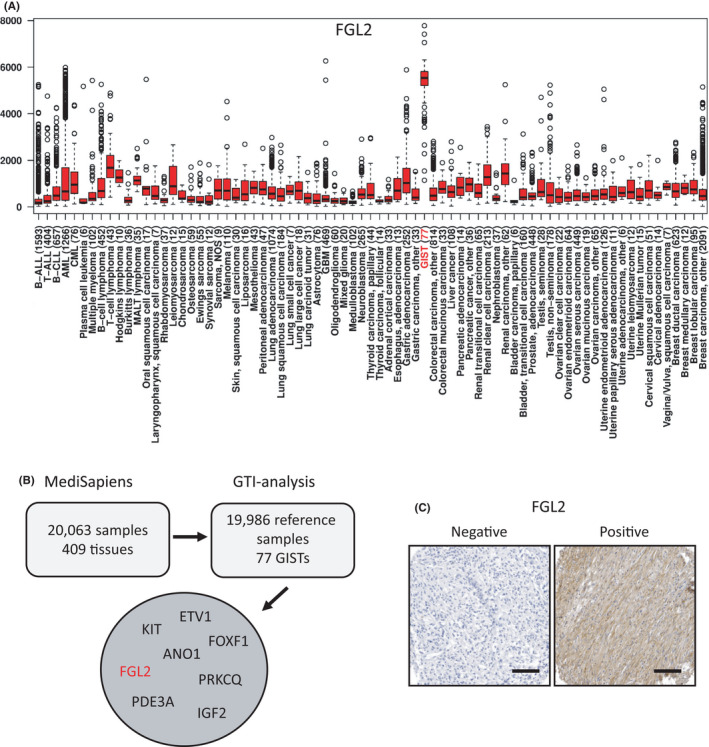
(A) A box‐whisker plot showing the relative *FGL2* mRNA expression in cancer. The bottom and the top of the box depict the 25th percentile and the 75th percentile, respectively, and the horizontal line the median. The whiskers extend to 1.5 times the interquartile range from the edges of the box, and the data points beyond the whiskers are marked with hollow circles. The number of samples studied is indicated in the brackets (modified from IST Online, ist.medisapiens.com). (B) GIST signature genes in the top 100 outlier list of the gene tissue index (GTI) analysis. mRNA expression profiles of GISTs were ranked using the GTI outlier statistics using the MediSapiens IST Online database. (C) Representative immunohistochemical stainings of FGL2‐positive and FGL2‐negative GIST tissue samples (magnification ×200; scale bar, 100 μm)

The IST Online database of human transcriptomes was first interrogated for genes that are highly expressed in GIST as compared with other cancers or normal tissues. According to the database, many types of cancer express low *FGL2* mRNA levels, but high *FGL2* expression is characteristic for GIST (Figure 1A). Blood monocytes and granulocytes, dendritic cells, connective tissues and adult stem cells also express *FGL2* mRNA (Figure S3). Besides *FGL2*, the list of the top 100 outlier genes that are highly expressed in GIST included *KIT*, *ANO1*, *ETV1*, *FOXF1*, *IGF2*, *PDE3A* and *PRKCQ* (Figure 1B, Table S4). Unlike *FGL2* mRNA expression, *FGL1* mRNA expression was low in GISTs (data not shown).

Expression of the FGL2 protein was next investigated using immunohistochemistry on a cancer tissue microarray that consisted of 36 human tumour types originating from 598 individuals. In an agreement with the *FGL2* mRNA analysis, FGL2 protein was detected frequently in GISTs. Thirty‐five (63%) out of the 56 GISTs examined stained for FGL2 (Figure 1C), whereas only two of the out of the 542 non‐GIST tumours consisting of 35 histological types stained for FGL2 (one meningioma and one schwannoma), suggesting that FGL2 expression is higher in GISTs than in most other types of human cancer (Table S5). The interstitial cells of Cajal expressed FGL2 unlike the bowel muscles or the fibroblast‐like PDGFRA‐positive cells that reside between the smooth muscle layers close to the interstitial cells of Cajal (Figure S4). Intratumoural leukocytes expressed FGL2.

### 3.2 FGL2 protein expression and GIST features

The clinical significance of GIST FGL2 expression was investigated in two large GIST patient series using immunohistochemistry, the western Sweden series patient population of whom none received adjuvant imatinib after surgery, and the SSGXVIII clinical trial series. FGL2 was frequent expressed in GISTs in both series, in 120 (78%) out of the 153 evaluable GISTs in the western Sweden series and in 213 (78%) out of the 272 evaluable GISTs in the SSGXVIII series. Thus, FGL2 protein was expressed in 333 (78%) out of the 425 GISTs investigated. When present, FGL2 was expressed both on the plasma membrane and in the cytoplasm of GIST cells.

The intrarater and inter‐rater agreement in the interpretation of the immunohistochemistry staining for FGL2 were studied between the 2 independent raters using tissue microarray GIST samples. The intrarater agreement was almost perfect (Cohen's kappa coefficient 0.83; 95% confidence interval [CI]: 0.80–0.86), and the interrater agreement was substantial (kappa coefficient 0.76; 95% CI: 0.69–0.83).

FGL2 expression was associated with favourable clinical features in both series, although the clinical and histopathological parameters available for evaluation differed somewhat between the series. In the western Sweden series, FGL2 was associated with a small GIST size at the time of the diagnosis, the spindle cell‐type morphology, the absence of tumour necrosis, a low mitotic count and a low or intermediate estimated risk of recurrence according to the National Institutes of Health stratification scheme (Table 1). No significant association was found with gender, age at the time of the diagnosis or tumour location in the gastrointestinal tract. In an agreement with these findings, GIST FGL2 expression was significantly associated with a low tumour mitotic count and small GIST size in the SSGXVIII series, whereas no association was found with the gender, age at the time of the diagnosis, tumour site or *KIT* or *PDGFRA* mutation (Table 2).

**TABLE 1:** Associations between GIST FGL2 expression and eight clinicopathological factors in the western Sweden series

Factor	GIST FGL2 expression		*p*
	Negative *N* = 33 *n* (%)	Positive *N* = 120 *n* (%)	
Gender			
Male	17 (24.3)	53 (75.7)	
Female	16 (19.3)	67 (80.7)	0.453
Site			
Gastric	16 (19.3)	67 (80.7)	
Nongastric	17 (24.3)	53 (75.7)	0.453
NIH risk stratification			
Low/intermediate	16 (15.5)	87 (84.5)	
High	17 (34.0)	33 (66.0)	0.009
Histological type			
Spindle cell	18 (16.5)	91 (83.5)	
Other	13 (33.3)	26 (66.7)	0.027
N.A.	2	3	
Tumour necrosis			
Absent	11 (14.7)	64 (85.3)	
Present	12 (33.3)	24 (66.7)	0.023
N.A.	10	32	
Mitotic count (per 50 HPFs)			
0–5	19 (16.2)	98 (83.8)	
>5	12 (38.7)	19 (61.3)	0.006
N.A.	2	3	
Median age, years (range)	68 (46–83)	69 (30–92)	0.619
Median tumour size, cm (range)	9.7 (1.0–30.0)	6.7 (0.5–33.0)	0.029

Abbreviations: HPF, high‐power field of the microscope; N.A., not available; NIH, the National Institutes of Health.

**TABLE 2:** Associations between GIST FGL2 expression and seven clinicopathological factors in the SSGXVIII trial patient population

Factor	GIST FGL2 expression		*p*
	Negative *N* = 59 *n* (%)	Positive *N* = 213 *n* (%)	
Gender			
Male	33 (24.6)	101 (75.4)	
Female	26 (18.8)	112 (81.2)	0.247
Site			
Gastric	26 (17.6)	122 (82.4)	
Nongastric	32 (26.2)	90 (73.8)	0.085
N.A.	1	1	
Tumour rupture			
Yes	10 (17.2)	48 (82.8)	
No	49 (22.9)	165 (77.1)	0.354
*KIT* or *PDGFRA* mutation			
*KIT* mutation	43 (20.3)	169 (79.7)	
*KIT* exon 11	41	150	
*KIT* exon 9	1	16	
*KIT* exon 13	1	3	
*PDGFRA* mutation	8 (25.8)	23 (74.2)	
*PDGFRA* D842V	5	18	
Neither *KIT n*or *PDGFRA* mutation	8 (36.4)	14 (63.6)	0.199
N.A.	0	7	
Mitotic count (per 50 HPFs)			
≤Median (≤6/50 HPFs)	19 (13.6)	121 (86.4)	
>Median (>6/50 HPFs)	37 (29.6)	88 (70.4)	0.001
N.A.	3	4	
Median age, years (range)	58 (22–79)	61 (26–81)	0.264
Median tumour size, cm (range)	11 (3–21)	9 (2–30)	0.009

Abbreviations: HPF, high‐power field of the microscope; N.A., not available.

### 3.3 Tumour‐infiltrating leucocyte counts

When GIST cell FGL2 expression was compared with tumour‐infiltrating leukocyte counts in the SSGXVIII series, FGL2 expression was significantly associated with a low tumour infiltrating CD3+ lymphocyte count and a low FoxP3+ lymphocyte count (*p* = 0.016 and.041 respectively), and tended to be associated with a small CD8+ count and a low CD20+ count (*p* = 0.065 and 0.051 respectively), whereas no association was found with the CD68+ macrophage count or the NCR1+ cell count (Table 3).

**TABLE 3:** Associations between GIST FGL2 expression and tumour‐infiltrating lymphocyte counts in the SSGXVIII series

Lymphocyte antigen	GIST FGL2 expression^a^		*p*
	Negative median count (range)	Positive median count (range)	
CD3+	131.7 (4.7–1679.5)	91.3 (0.0–1369.5)	0.016
CD8+	66.6 (0.0–387.7)	40.4 (0.0–1186.3)	0.065
CD20+	26.1 (0.0–228.6)	10.3 (0.0–1311.1)	0.051
CD68+	176.6 (38.6–622.6)	153.0 (25.9–1387.9)	0.268
FoxP3+	21.8 (0.0–168.7)	15.1 (0.0–471.9)	0.041
NCR1+	0.0 (0.0–68.5)	0.0 (0.0–255.6)	0.576

Abbreviation: SSG, Scandinavian Sarcoma Group.

^a^ Out of the 272 patients with FGL2 data available in the series, 32, 52, 54, 73, 66 and 52 patients had missing data for CD3+, CD8+, CD20+, CD68+, FoxP3+ and NCR1+ analyses respectively.

### 3.4 FGL2 expression and survival

**FIGURE 2:**
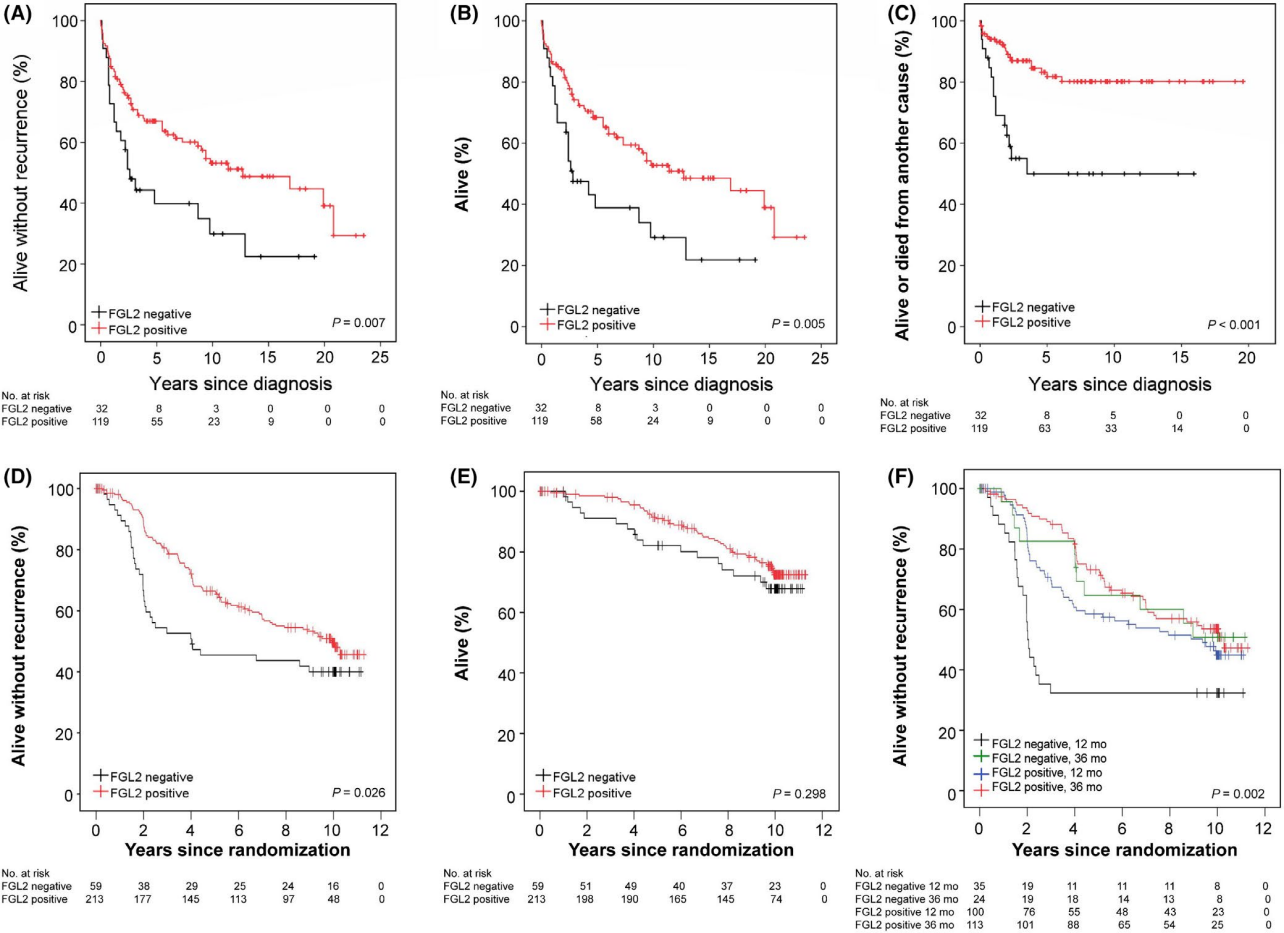
Associations between GIST FGL2 protein expression with recurrence‐free survival (A), overall survival (B), and GIST‐specific survival (C) in the western Sweden population‐based series. Associations between GIST FGL2 protein expression with recurrence free survival (D), overall survival (E) and recurrence‐free survival stratified by the duration of adjuvant imatinib (either 12 months or 36 months) (F) in the SSGXVIII trial series. The patients censored are indicated with a bar

Patients whose GIST expressed FGL2 had more favourable survival outcomes than patients whose GIST was FGL2 negative both in the western Sweden series and the SSGXVIII series. In the western Sweden series, patients whose GIST expressed FGL2 had longer RFS than patients whose GIST did not express FGL2 (hazard ratio [HR] =0.51; 95% CI: 0.31–0.84; log‐rank test *p* = 0.007), longer overall survival (HR =0.50; 95% CI: 0.30–0.82; *p* = 0.005), and longer GIST‐specific survival (HR = 0.30; 95% CI: 0.15–0.60; *p* < 0.001) in univariable survival analyses (Figure 2A‐C). When FGL2 expression (positive vs negative) was entered as a covariable into a Cox multivariable analysis together with tumour mitotic count (⩽5 vs. >5 mitoses/50 HPFs), tumour size (as a continuous variable) and site (gastric vs nongastric) using RFS as the end point, FGL2 expression (HR = 0.56; 95% CI: 0.32–0.96; *p* =.036), low mitotic count (HR =0.25; 95% CI: 0.14–0.43; *p* < 0.001) and small tumour size (HR =0.93; 95% CI: 0.92–0.95; *p* < 0.001) were significantly associated with favourable RFS, whereas tumour site was not (HR = 0.90; 95% CI: 0.63–1.28; *p* =.555).

In accordance with the western Sweden series, patients with FGL2‐positive GIST had superior RFS as compared to those whose GIST did not express FGL2 in the SSGXVIII series (HR = 0.64, 95% CI: 0.44–0.95; *p* = 0.026), but there was no significant association between GIST FGL2 expression and overall survival (*p *= 0.298; Figure 2D‐E). Interestingly, in the subgroup of patients who were randomly allocated to receive adjuvant imatinib for 36 months, GIST FGL2 expression had little influence on RFS (HR = 0.92, 95% CI: 0.48–1.77), whereas in the subgroup of patients allocated to receive imatinib for 12 months, patients with FGL2‐positive GIST had substantially better RFS as compared to those with FGL2‐negative GIST (HR = 0.52, 95% CI 0.32–0.86, Figure 2F). The interaction term between GIST FGL2 expression and the adjuvant treatment duration was, however, not significant (*p* = 0.172). Unlike in the western Sweden series, GIST FGL2 expression did not have independent prognostic value when entered as a covariable into a Cox's multivariable analysis together with tumour mitotic count (as a continuous variable), tumour size (as a continuous variable), tumour site (gastric vs nongastric), tumour rupture (no vs yes) and adjuvant imatinib duration (12 vs. 36 months), whereas each of the other covariables were significantly associated with RFS (*p *< 0.01 for each covariable).

### 3.5 Effect on GIST cell signalling

**FIGURE 3:**
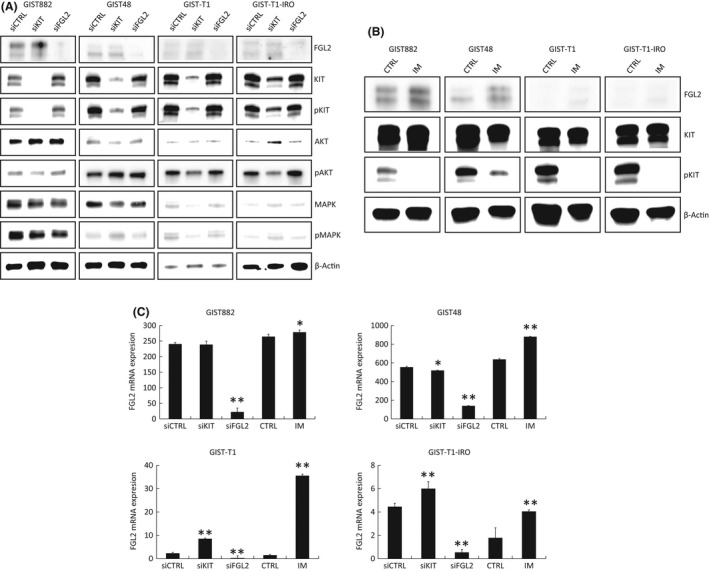
(A) A western plot showing expression of FGL2, KIT, phosphorylated KIT (Y719), AKT, phosphorylated AKT (S473), MAPK, phosphorylated MAPK (T202/Y204) and β‐actin (control) in the GIST882, GIST48, GIST‐T1 and GIST‐T1‐IRO cell lines. Expression 72 hours after *KIT* and *FGL2* siRNA transfection are shown. (B), A western blot showing the FGL2, KIT, phosphorylated KIT (Y719) and β‐actin (control) expression in the GIST882, GIST48, GIST‐T1 and GIST‐T1‐IRO cell lines 72 hours after imatinib (1 μmol/L) treatment. (C) Normalized *FGL2* mRNA expression measured with qPCR from the GIST cell lines 72 hours after *KIT* and *FGL2* siRNA transfection or imatinib (1 μmol/L) treatment. *p < 0.05, and **p <.001. The *P* values refer to the comparisons with the controls (CTRL). Data represent the mean ±the standard error of mean (SEM). A G:BOX Chemi XX9 imaging system was used for western blot imaging (A, B) leading to clear white backgrounds

All four GIST cell lines studied expressed FGL2. The GIST48 cells had the highest expression followed by GIST882, and GIST‐T1 and GIST‐T1‐IRO cells had the lowest expression. *KIT* inhibition with siRNA tended to induce FGL2 expression in the cell lines, notably in the GIST‐T1 and GIST‐T1‐IRO cell lines that had the lowest basal expression (Figure 3). Imatinib treatment increased FGL2 expression levels significantly in all cell lines, and in three out of the four cell lines more than *KIT* inhibition with siRNA. *FGL2* attenuation had no effect on KIT or phospho‐KIT expression, or on the KIT downstream signalling molecules AKT kinase, pAKT, mitogen‐activated protein kinase (MAPK) or pMAPK.

### 3.6 Cell viability

**FIGURE 4:**
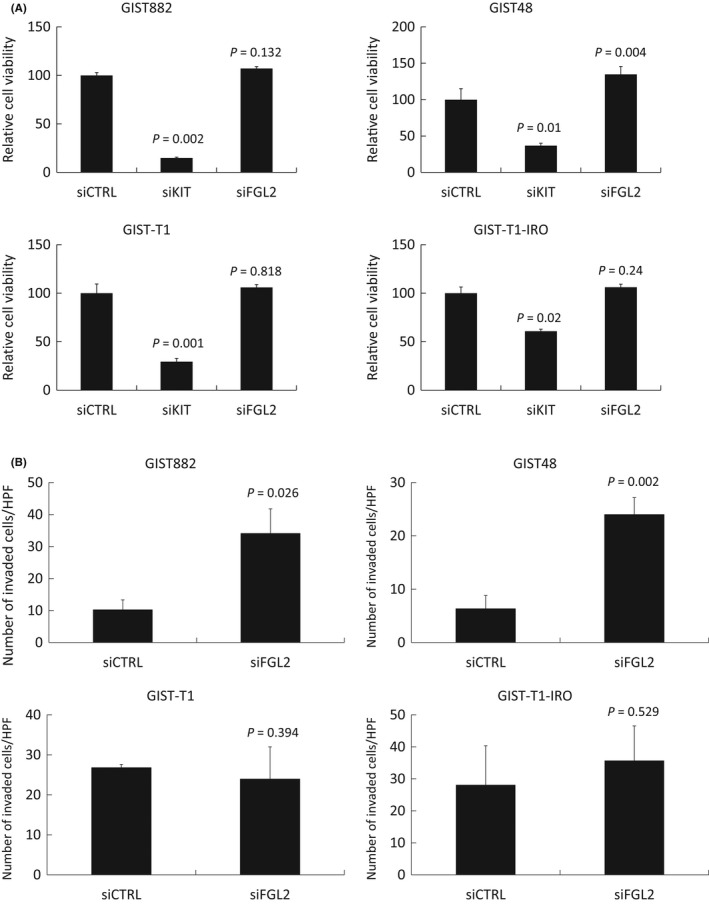
Effect of transfection with control siRNA, *KIT* siRNA and *FGL2* siRNA on cell viability (A) and invasion (B) in GIST882, GIST48, GIST‐T1 and GIST‐T1 cell lines. (A) *KIT* siRNA transfection decreased cell viability in all cell lines, *FGL2* siRNA in none of them. (B) *FGL2* siRNA transfection increased cell invasion in 2 out of the 4 cell lines investigated (GIST882 and GIST48) 24 hours after transfection. The bars indicate the average number of invaded cells ± the standard error of mean (SEM) per 1 microscope high power field (HPF)

Silencing of *FGL2* with siRNA had relatively little effect on cell viability in the GIST48, GIST882, GIST‐T1 and GIST‐T1‐IRO cell lines, whereas silencing of *KIT* with siRNA decreased cell viability substantially (Figure 4A). Cell viability decreased less with *KIT* siRNA in the imatinib‐resistant GIST‐T1‐IRO cell line compared to the parental GIST‐T1 cell line. *FGL2* mRNA knockdown with siRNA increased the viability of the GIST48 cells and reduced the effect of imatinib slightly, whereas *FGL2* silencing had little effect on cell viability in the other three cell lines (Figure S5 and Figure S6).

### 3.7 Cell invasion

The influence of *FGL2* downregulation on GIST cell invasion was evaluated in a matrigel‐coated transwell system. The number of invaded GIST882 and GIST48 cells increased substantially after *FGL2* silencing with siRNA as compared with the control siRNAs, whereas a similar effect was not observed with the more invasive GIST‐T1 and GIST‐T1‐IRO cell lines (Figure 4B).

## 4 DISCUSSION

We found that FGL2, a fibrinogen‐related protein, is expressed frequently in GISTs as compared with many other human cancers, and it is expressed also in the interstitial cells of Cajal. FGL2 expression was associated with favourable RFS in two large GIST patient series. In an agreement with the survival outcomes, FGL2 expression was associated with several favourable risk factors for GIST recurrence, such as a low cell proliferation rate, small size and the absence of tumour necrosis, and also with low TIL counts. Interfering with FGL2 expression using siRNA increased invasiveness of two GIST cell lines. Interestingly, inhibiting KIT with imatinib increased FGL2 expression in the investigated GIST cell lines.

We found in the randomized SSGXVIII trial patient population that patients with FGL2‐negative GIST had particularly poor RFS in the 1‐year adjuvant imatinib group but not in the 3‐year group, suggesting that patients with FGL2‐negative GIST might benefit most from the 3‐year duration of adjuvant imatinib. Since imatinib treatment upregulated FGL2 expression in the GIST cell lines studied, one could speculate that prolonged adjuvant imatinib treatment might upregulate FGL2 also in patients. The interaction term between GIST FGL2 expression and the duration of adjuvant imatinib treatment was not statistically significant, but the analysis to detect the interaction was likely underpowered, since the numbers of patients with FGL2‐negative GIST were relatively small and the SSGXVIII trial was powered for testing of the two durations of adjuvant imatinib in a larger population of 400 GIST patients. Nevertheless, this finding needs to be viewed with caution because it is based on a retrospective analysis of a clinical trial.

The efficacy of immune checkpoint inhibitors has been limited in GISTs.^20^, ^21^ It is unknown whether frequent FGL2 expression in GIST hinders the clinical efficiency of checkpoint inhibitors. FGL2 mediates a wide variety of immunological effects and sFGL2 is immunosuppressive, and could, therefore, promote cancer growth.^9^, ^22^ In this study, FGL2 expression was associated with low numbers of tumour‐infiltrating CD3+, CD8+, CD20+ and Foxp3+ lymphocytes, which is in agreement with the immunosuppressive role of sFGL2.^9^ FGL2 binding to the FcγRIIB receptor enhances immunosuppression.^23^ However, a high number of CD3+, CD8+, CD45RO+, NKp46+ and CD20+ expressing cells has been linked with favourable survival in GIST,^24^ CD8+ T cells may contribute to the antitumor effects of imatinib,^23^ and imatinib may activate CD8+ T cells and induce tumour Treg apoptosis by reducing the expression of indoleamine 2,3‐dioxygenase.^25^ Taken together, these findings suggest that the host immune function may be of importance in the clinical behaviour of GIST. FGL2 may have also other functions in GIST besides immunomodulation, such as inhibition of cell invasion.

Previous studies on the role of FGL2 in other types of human cancer have produced seemingly inconsistent results. FGL2 has been found to enhance tumour cell proliferation, promote the coagulation cascade and induce angiogenesis.^9^ In line with these findings, high FGL2 expression was associated with poor prognosis in glioma and clear cell renal cell carcinoma.^26^, ^27^ *FGL2* knockout in glioma cells did not affect the proliferation of tumour cells in immunocompromised mice but completely impaired glioblastoma progression in immune‐competent mice, suggesting that FGL2 has an important role in regulation of the immune environment in glioblastoma.^28^ Depletion of FGL2 inhibited colorectal carcinoma progression and enhanced epithelial‐to‐mesenchymal transition *in vitro* and *in vivo*,^29^ and deficiency of host *FGL2* was associated with reduced growth of lung cancer.^30^ On the other hand, others have found that high expression of FGL2 is associated with favourable survival of patients with lung adenocarcinoma or breast cancer.^31^, ^32^ These variable results may be due the different functions of mFGL2 and sFGL2, which needs to be considered when assessing the effects of FGL2 in cancer. The biological functions of FGL2 may also vary in different types of human cancer.

The present study has some limitations. The quantitation of FGL2 protein expression using immunohistochemistry is subjective, but when the scores were compared between two independent observers the agreement turned out to be substantial. The FGL2 antibody used was polyclonal, increasing the possibility of unspecific antibody binding. The quality of tumour tissue used may also have influenced the results, and we cannot exclude the effect of factors such as variability in tissue sample fixation. However, the results from two large clinical GIST series investigated were well in agreement although only patients with high‐risk GIST were included in the SSGXVIII clinical trial.

## 5 CONCLUSIONS

We conclude FGL2 expression is high in GISTs compared to many other human cancers. FGL2 expression in GIST was associated with the low‐risk category of the National Institutes of Health risk stratification scheme, a low cell proliferation rate, small tumour size and favourable RFS in clinical patient series. High FGL2 expression in GISTs and its association with low TIL counts might explain in part the modest results obtained with immune checkpoint inhibitors in advanced GIST. Imatinib induced FGL2 expression in GIST cell lines, and patients with FGL2‐negative GIST had substantially longer RFS when treated with 3 years of adjuvant imatinib than when treated with 1 year of imatinib. The present results suggest that FGL2 influences the clinical behaviour of GIST.

## CONFLICT OF INTEREST

OPP, HS and HJ hold shares of Sartar Therapeutics. HJ is the Chair of the Scientific Advisory Board at Orion Pharma and at Neutron Therapeutics Ltd., PR reports an advisory role for Bayer, Clinigen, Roche, MSD, Deciphera, PharmaMar, Mundibiopharma and speaker's honoraria from Lilly and PharmaMar. ME is a consultant for Blueprint Medicines, and has participated in advisory boards for Clinigen and Bayer, ME is a trial physician in the Scandinavian Sarcoma Group, which receives support from Novartis. LV, ML, KSH, AR, EW and BN report no conflicts of interest.

## AUTHOR CONTRIBUTIONS

**Olli Pekka Pulkka:** Conceptualization (lead); Data curation (lead); Formal analysis (lead); Investigation (lead); Methodology (lead); Project administration (lead); Resources (lead); Supervision (lead); Validation (lead); Visualization (lead); Writing – original draft (lead); Writing – review & editing (lead). **Leevi Viisanen:** Data curation (supporting); Formal analysis (supporting); Investigation (supporting); Validation (supporting); Writing – original draft (supporting); Writing – review & editing (supporting). **Olli Tynninen:** Formal analysis (supporting); Investigation (supporting); Validation (supporting); Writing – original draft (supporting); Writing – review & editing (supporting). **Maria Laaksonen:** Formal analysis (supporting); Validation (supporting); Writing – original draft (supporting); Writing – review & editing (supporting). **Peter Reichardt:** Resources (supporting); Writing – original draft (supporting); Writing – review & editing (supporting). **Annette Reichardt:** Resources (supporting); Writing – original draft (supporting); Writing – review & editing (supporting). **Mikael Eriksson:** Resources (supporting); Writing – original draft (supporting); Writing – review & editing (supporting). **Kirsten Sundby Hall:** Resources (supporting); Writing – original draft (supporting); Writing – review & editing (supporting). **Eva Wardelmann:** Resources (supporting); Writing – original draft (supporting); Writing – review & editing (supporting). **Bengt Nilsson:** Resources (supporting); Writing – original draft (supporting); Writing – review & editing (supporting). **Harri Sihto:** Conceptualization (supporting); Data curation (supporting); Methodology (supporting); Supervision (supporting); Writing – original draft (supporting); Writing – review & editing (supporting). **Heikki Joensuu:** Conceptualization (lead); Data curation (lead); Formal analysis (equal); Funding acquisition (lead); Investigation (equal); Methodology (lead); Project administration (lead); Resources (lead); Software (lead); Supervision (lead); Validation (lead); Visualization (equal); Writing – original draft (lead); Writing – review & editing (lead).

## Supporting information

Fig S1

Fig S2

Fig S3

Fig S4

Fig S5

Fig S6

Table S1

Table S2

Table S3

Table S4

Table S5

## Data Availability

The data that support the findings of this study are available from the corresponding author upon reasonable request.
