# Supplementary material for: Fibrinogen‐like protein 2 in gastrointestinal stromal tumour
Source: J Cell Mol Med. 2022 Jan 14;26(4):1083–94. doi: 10.1111/jcmm.17163 (PMC8831987; doi:10.1111/jcmm.17163)
Supplement: Supplementary file 1 — Fig S1 [file JCMM-26-1083-s006.pdf]

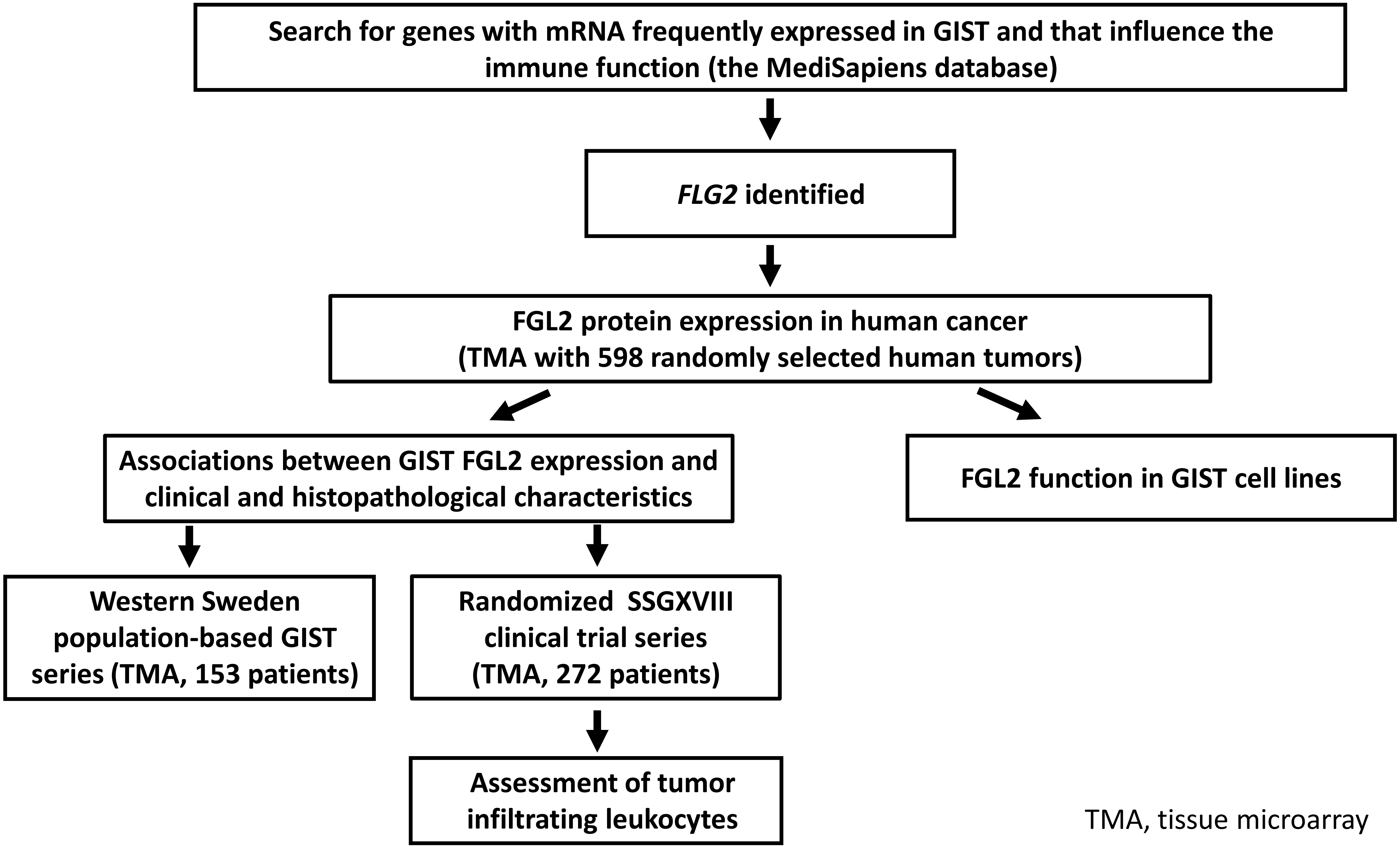
Search for genes with mRNA frequently expressed in GIST and that influence the immune function (the MediSapiens database)
FLG2 identified
FGL2 protein expression in human cancer (TMA with 598 randomly selected human tumors)
Associations between GIST FGL2 expression and clinical and histopathological characteristics
FGL2 function in GIST cell lines
Western Sweden population-based GIST series (TMA, 153 patients)
Randomized SSGXVIII clinical trial series (TMA, 272 patients)
Assessment of tumor infiltrating leukocytes
TMA, tissue microarray
