# Supplementary figures and images for: Fibrinogen‐like protein 2 in gastrointestinal stromal tumour
Source: J Cell Mol Med. 2022 Jan 14;26(4):1083–94. doi: 10.1111/jcmm.17163 (PMC8831987; doi:10.1111/jcmm.17163)

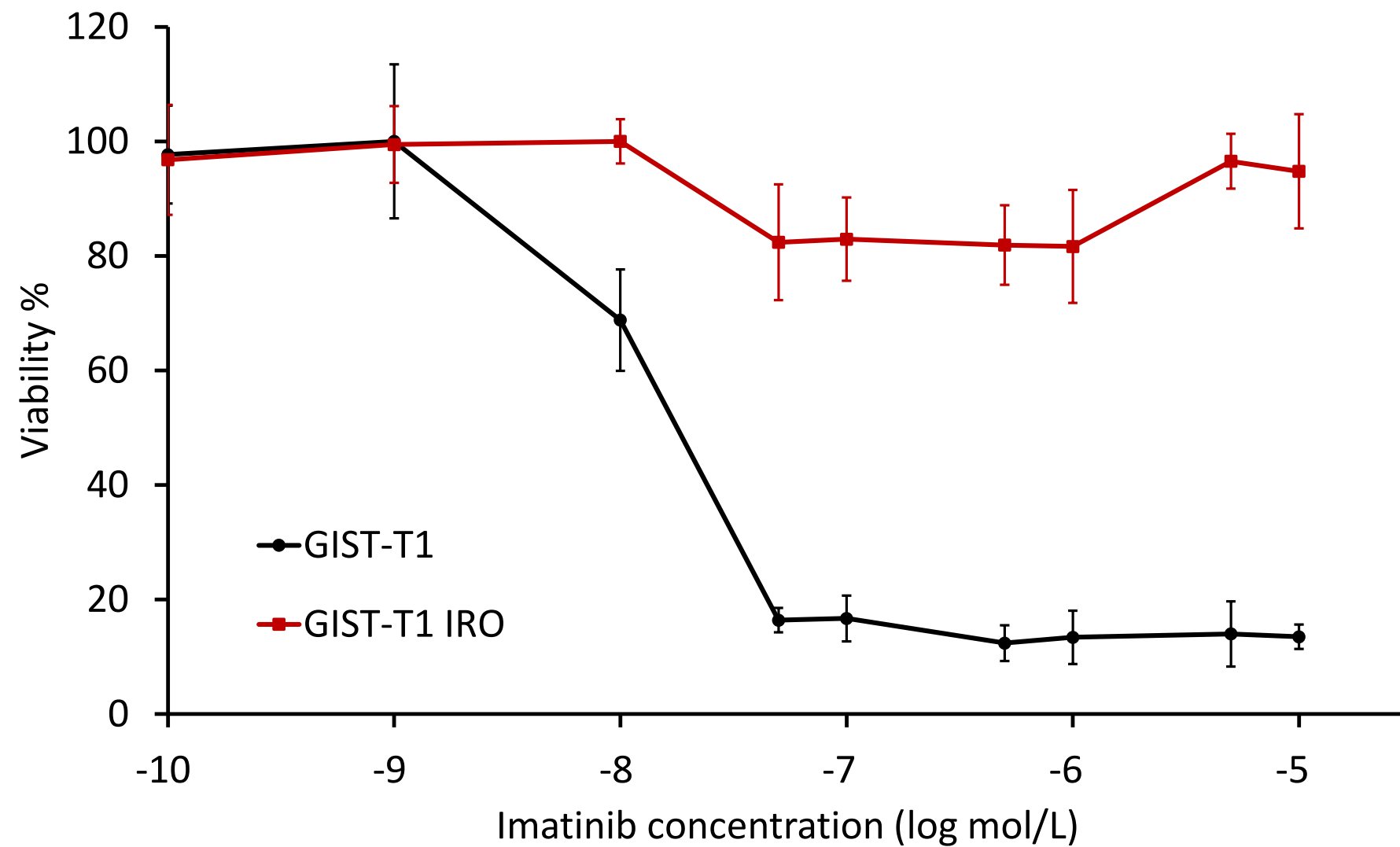

Supplement: Supplementary file 2 — Fig S2 [file JCMM-26-1083-s005.pdf]

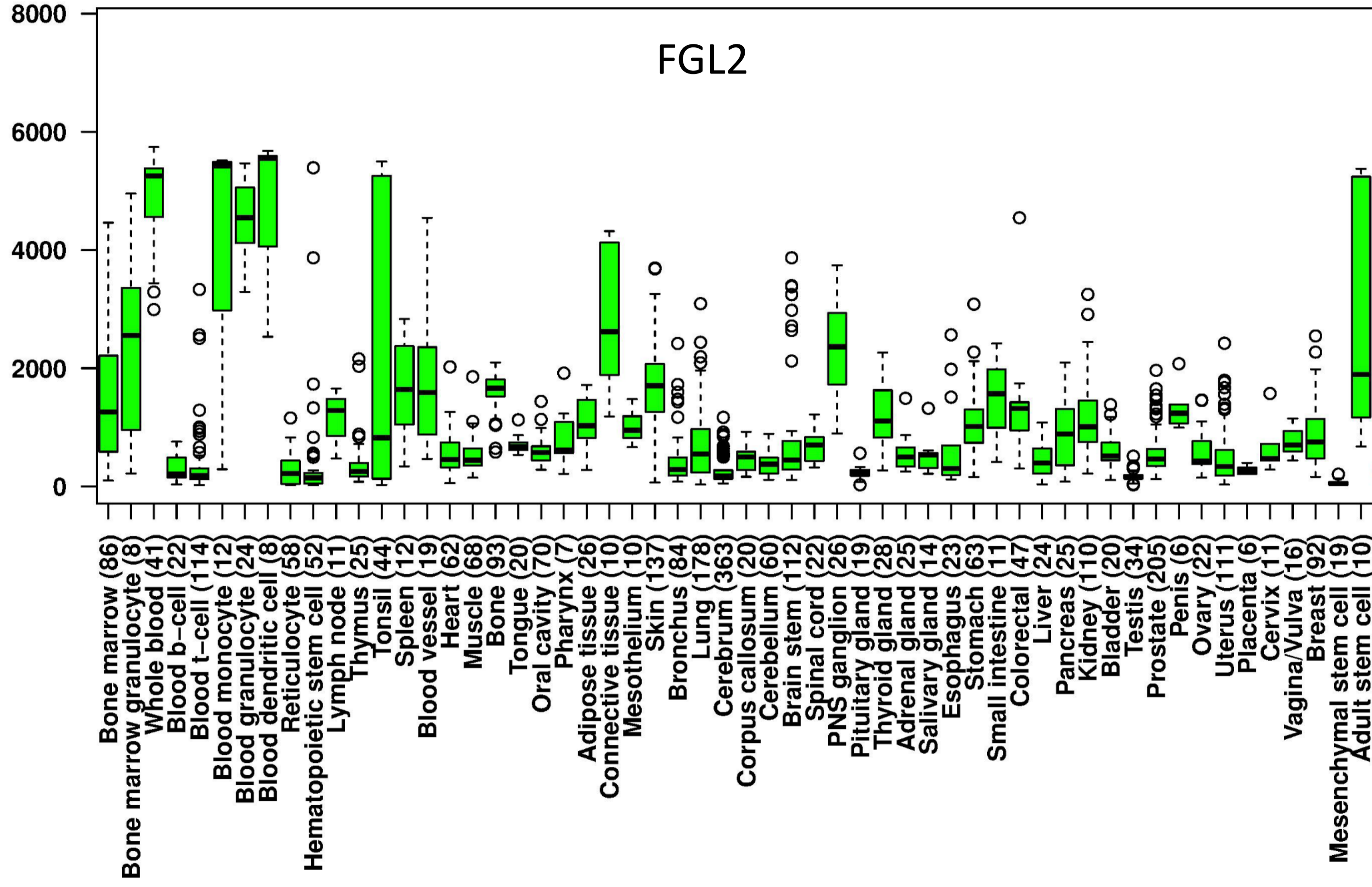

Supplement: Supplementary file 3 — Fig S3 [file JCMM-26-1083-s004.pdf]

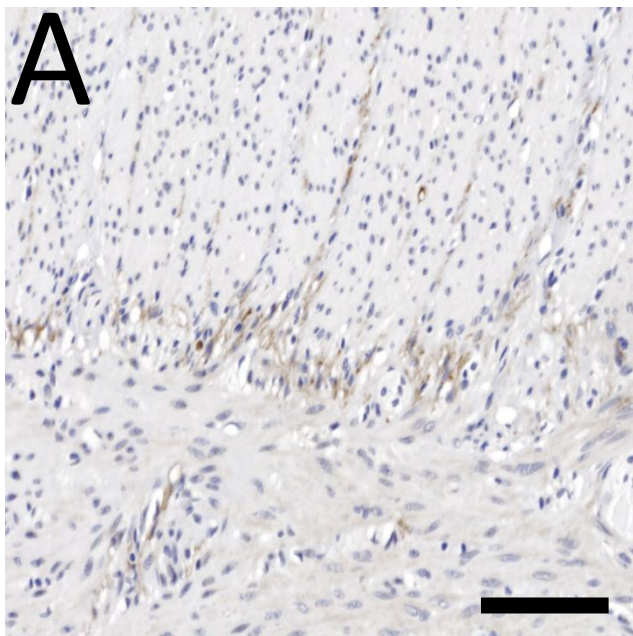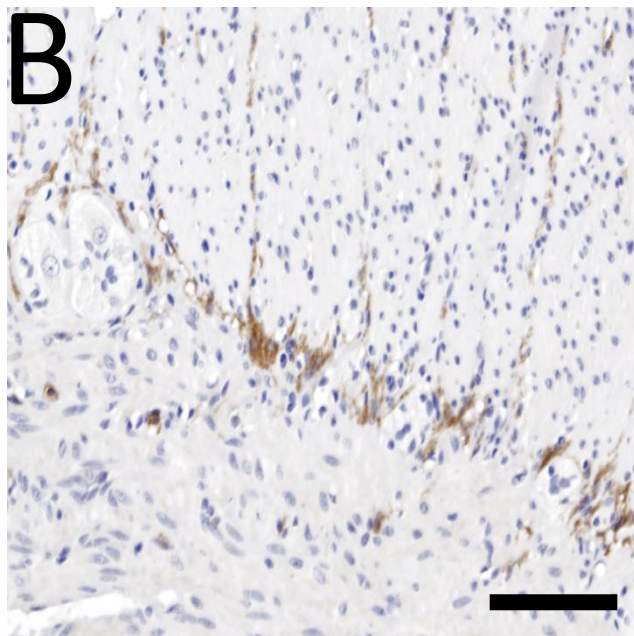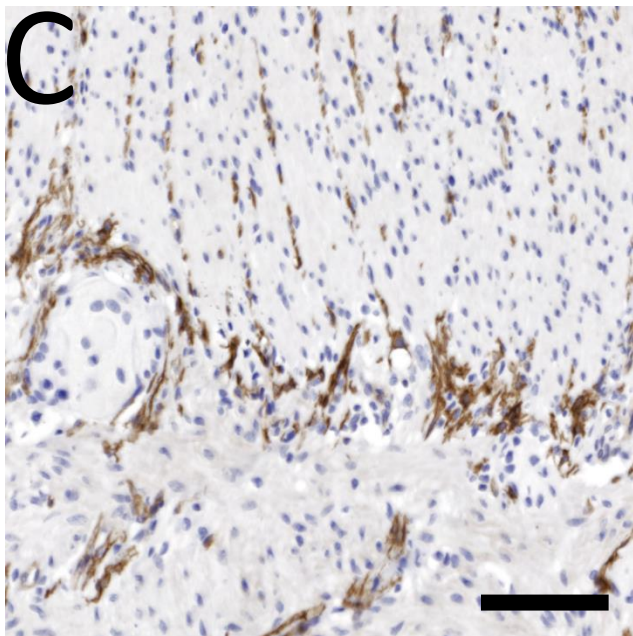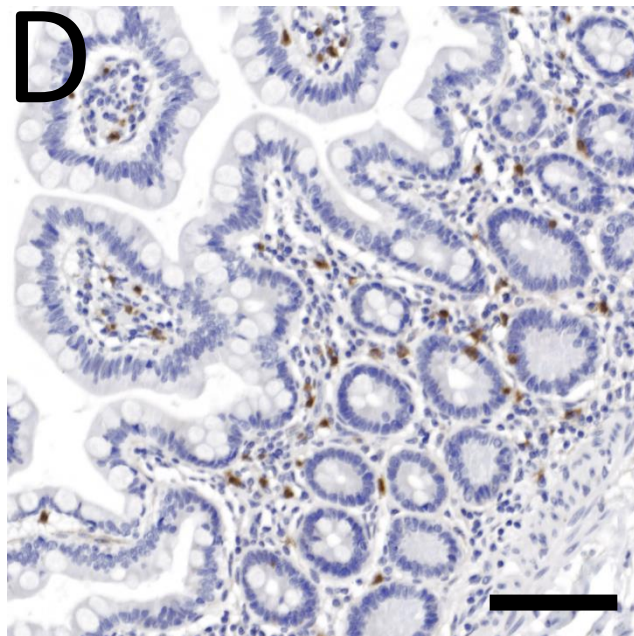

Supplement: Supplementary file 4 — Fig S4 [file JCMM-26-1083-s003.pdf]

GIST882

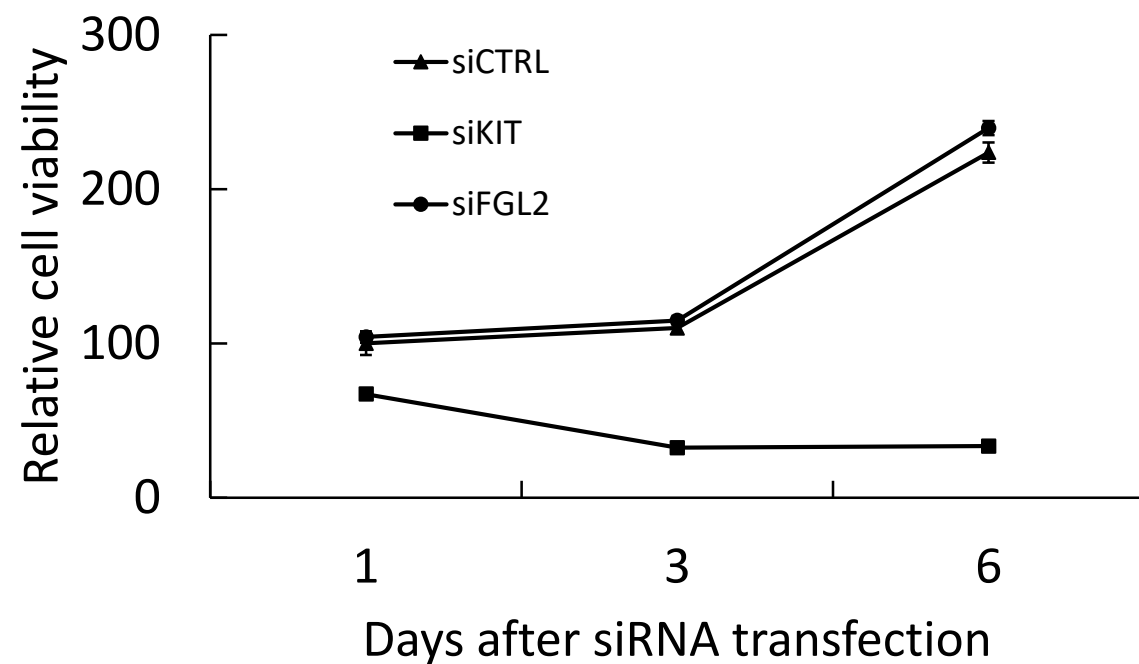

GIST48

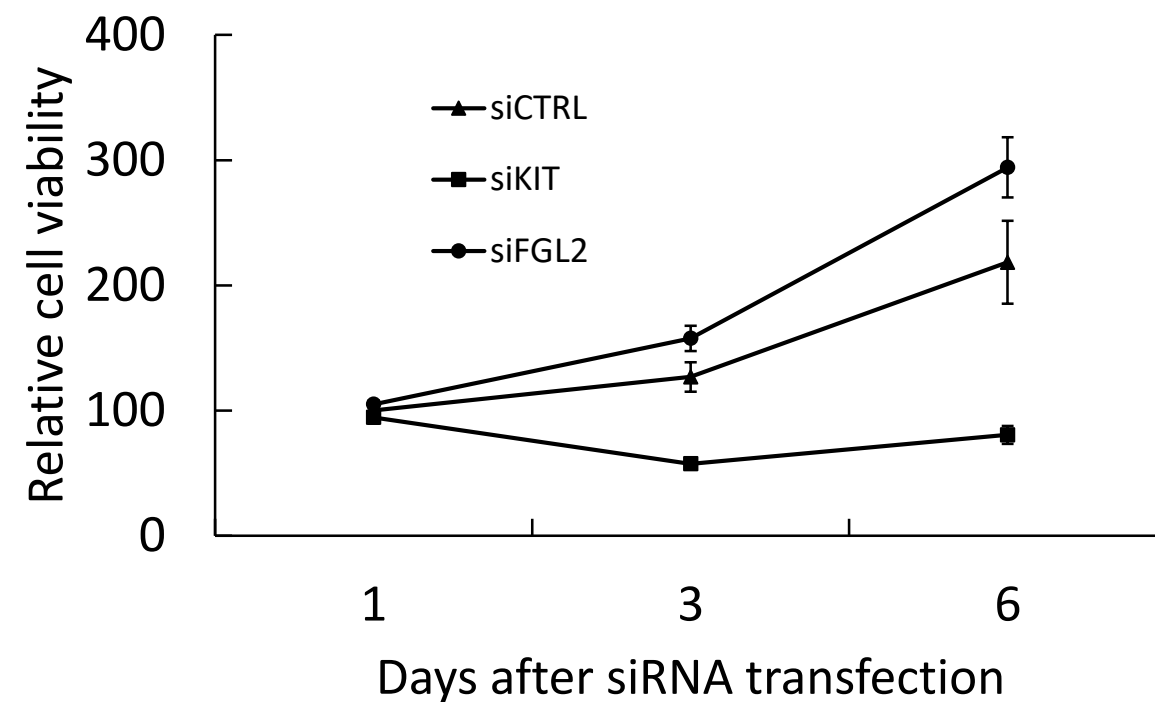

GIST-T1

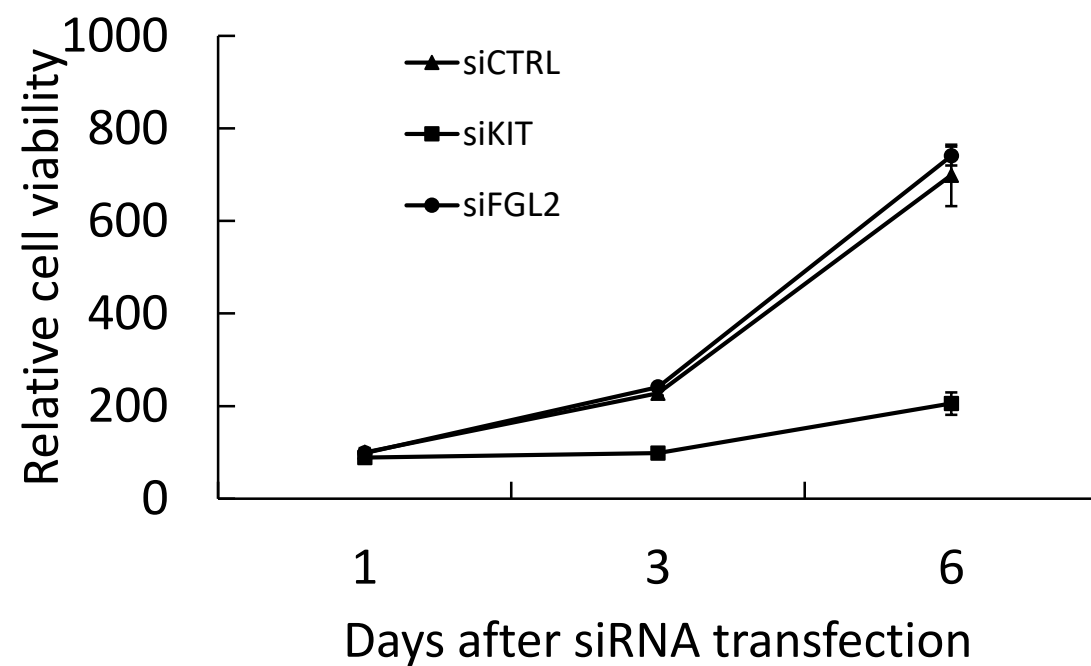

GIST-T1-IRO

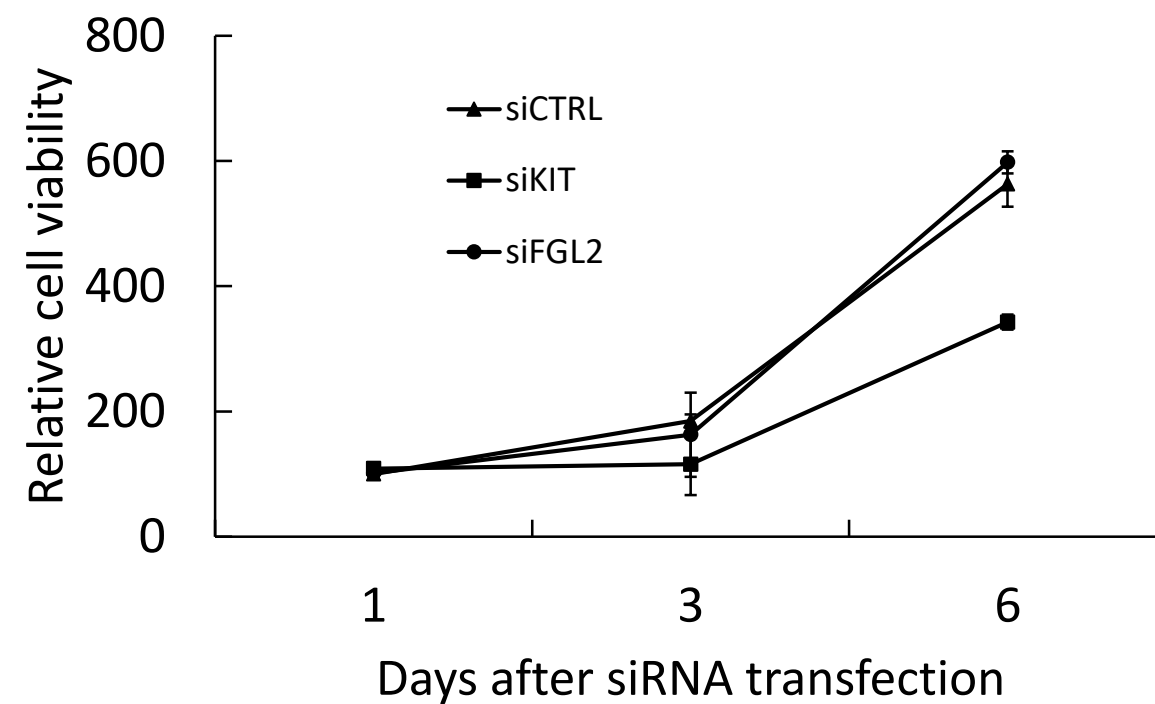

Supplement: Supplementary file 5 — Fig S5 [file JCMM-26-1083-s001.pdf]

GIST882

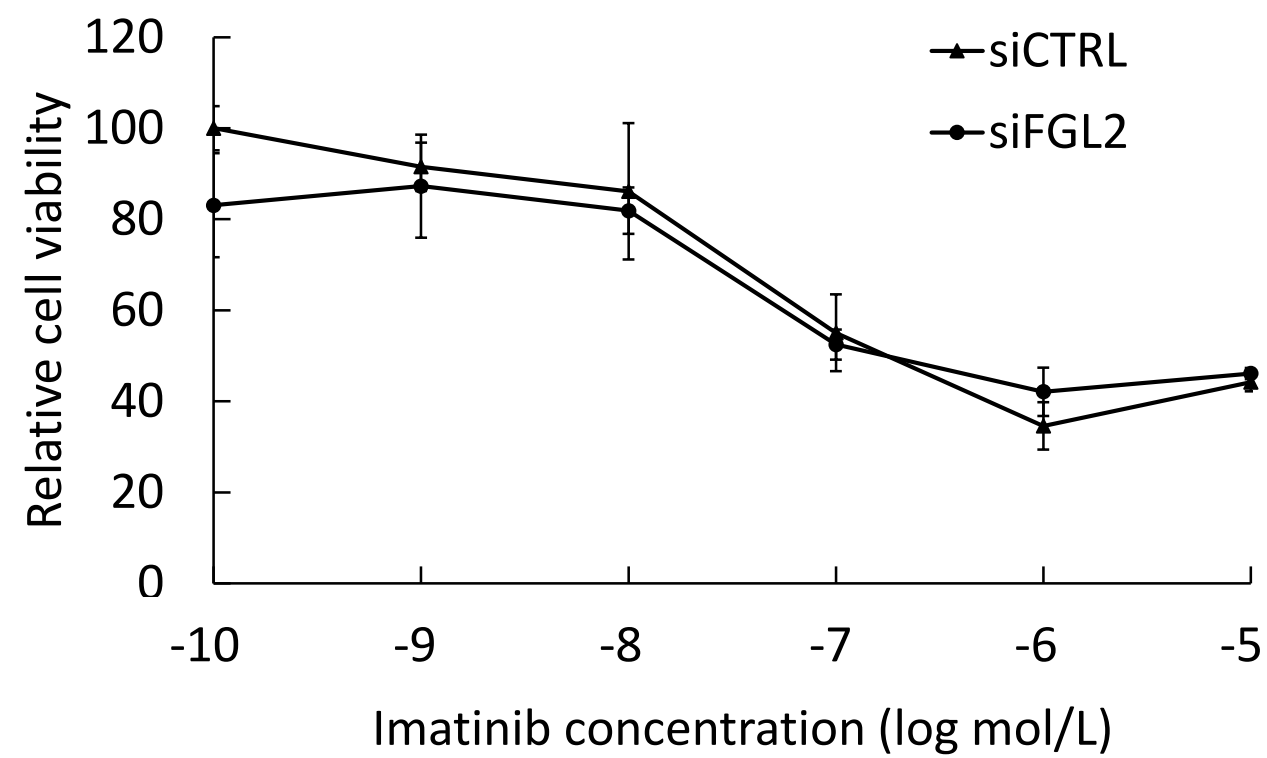

GIST48

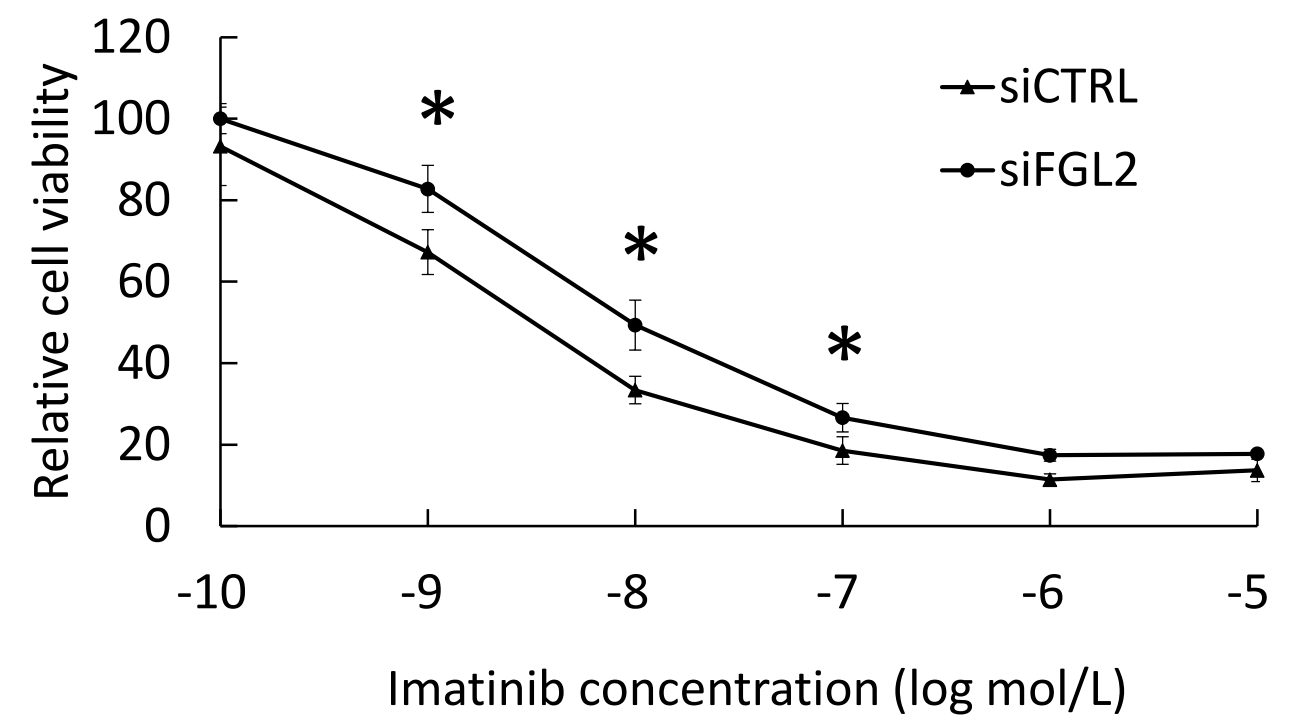

GIST-T1

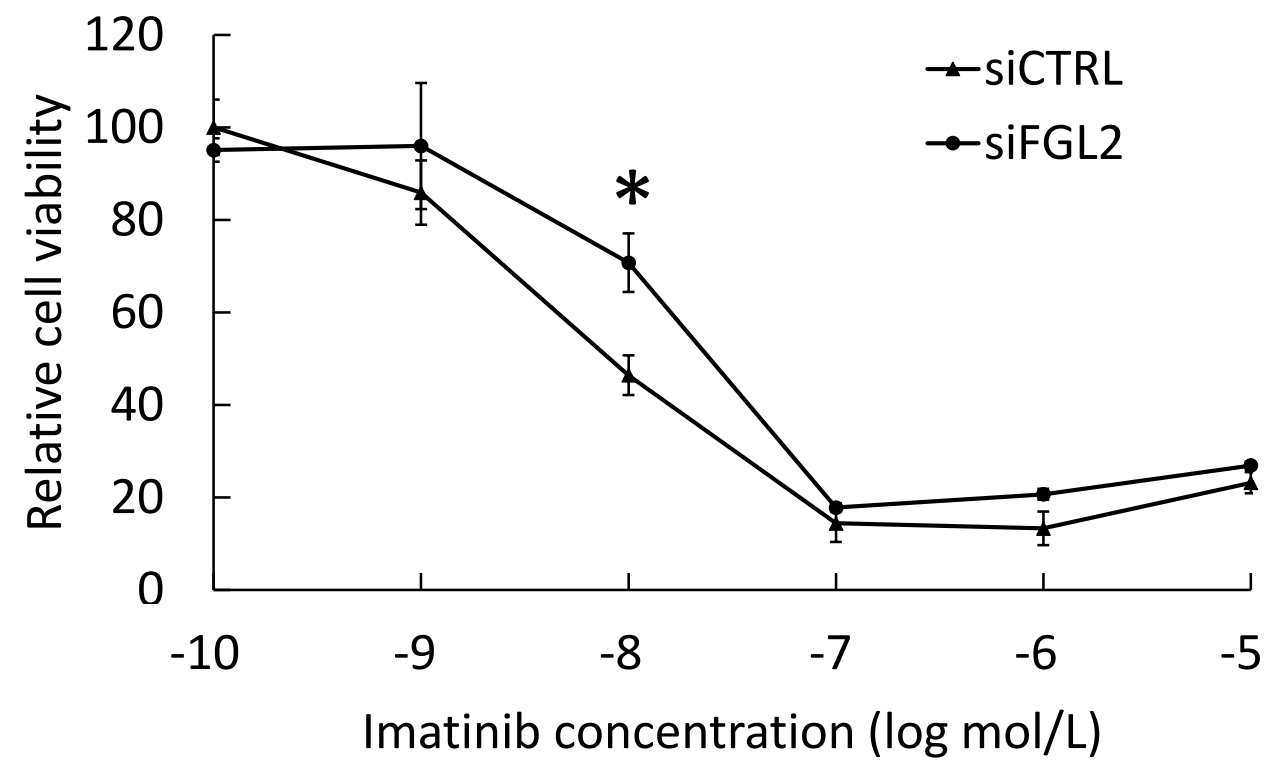

Supplement: Supplementary file 6 — Fig S6 [file JCMM-26-1083-s009.pdf]
