# Supplementary material for: Fibrinogen‐like protein 2 in gastrointestinal stromal tumour
Source: J Cell Mol Med. 2022 Jan 14;26(4):1083–94. doi: 10.1111/jcmm.17163 (PMC8831987; doi:10.1111/jcmm.17163)
Supplement: Supplementary file 8 — Table S2 [file JCMM-26-1083-s011.docx]

**Supplementary Table 2.** Antibodies Used in Automated Immunohistochemistry

| Target | Antibody | Origin, class | Incubation time | Dilution | Manufacturer |
| --- | --- | --- | --- | --- | --- |
| CD3 | sc-137096 | Mouse, monoclonal | 1 h at RT | 1:100 | Santa Cruz Biotechnology |
| CD8 | MA5-14548 | Rabbit, monoclonal | 30 min at RT | 1:100 | Thermo Scientific |
| CD20 | sc-58985 | Mouse, monoclonal | 2 h at RT | 1:200 | Santa Cruz Biotechnology |
| CD68 | sc-20060 | Mouse, monoclonal | 30 min at RT | 1:500 | Santa Cruz Biotechnology |
| FOXP3 | Ab20034 | Mouse, monoclonal | Overnight at +4°C | 1:300 | Abcam |
| NCR1 | Ab14823 | Mouse, monoclonal | 1 h at RT | 1:700 | Abcam |
| KIT | sc-168 | Rabbit, polyclonal | Overnight at +4°C | 1:50 | Santa Cruz Biotechnology |

RT, room temperature.
