# Supplementary material for: Fibrinogen‐like protein 2 in gastrointestinal stromal tumour
Source: J Cell Mol Med. 2022 Jan 14;26(4):1083–94. doi: 10.1111/jcmm.17163 (PMC8831987; doi:10.1111/jcmm.17163)
Supplement: Supplementary file 9 — Table S3 [file JCMM-26-1083-s002.docx]

**Supplementary Table 3.** Antibodies Used in Western Blotting

| Target | Antibody | Origin, class | Dilution | Manufacturer |
| --- | --- | --- | --- | --- |
| β-Actin | A300-491A | Rabbit, polyclonal | 1:10000 | Bethyl Laboratories |
| AKT | sc-5298 | Mouse, monoclonal | 1:1000 | Santa Cruz Biotechnology |
| pAKT (S473) | 4060 | Rabbit, monoclonal | 1:1000 | Cell signaling technologies |
| FGL2 | HPA021011 | Rabbit, polyclonal | 1:1000 | Sigma |
| KIT | A4502 | Rabbit, polyclonal | 1:10000 | DAKO |
| p-KIT (Y719) | 3391 | Rabbit, polyclonal | 1:10000 | Cell signaling technologies |
| MAPK | 9102 | Rabbit, polyclonal | 1:1000 | Cell signaling technologies |
| pMAPK (T202/Y204) | 4370 | Rabbit, monoclonal | 1:1000 | Cell signaling technologies |
