# Supplementary material for: Fibrinogen‐like protein 2 in gastrointestinal stromal tumour
Source: J Cell Mol Med. 2022 Jan 14;26(4):1083–94. doi: 10.1111/jcmm.17163 (PMC8831987; doi:10.1111/jcmm.17163)
Supplement: Supplementary file 11 — Table S5 [file JCMM-26-1083-s010.docx]

**Supplementary Table 5.** FGL2 Expression in 598 Human Tumors

| Tumor type | No. of tumors studied | FGL2 expression positive n (%) |
| --- | --- | --- |
| Gastrointestinal stromal tumor | 56 | 35 (63) |
| Other sarcomas |  |  |
| Angiosarcoma | 13 | 0 (0) |
| Chondrosarcoma | 5 | 0 (0) |
| Fibrosarcoma | 5 | 0 (0) |
| Undifferentiated pleomorphic sarcoma | 38 | 0 (0) |
| Leiomyosarcoma | 30 | 0 (0) |
| Liposarcoma | 42 | 0 (0) |
| Synovial sarcoma | 16 | 0 (0) |
| Central nervous system tumor |  |  |
| Astrocytoma | 8 | 0 (0) |
| Glioblastoma | 47 | 0 (0) |
| Medulloblastoma | 18 | 0 (0) |
| Meningioma | 8 | 1 (13) |
| Oligodendroglioma | 10 | 0 (0) |
| Schwannoma | 4 | 1 (25) |
| Breast cancer |  |  |
| Ductal | 65 | 0 (0) |
| Lobular | 3 | 0 (0) |
| Cholangiocarcinoma | 2 | 0 (0) |
| Colon, adenocarcinoma | 6 | 0 (0) |
| Corpus uteri, adenocarcinoma | 7 | 0 (0) |
| Hepatocellular carcinoma | 21 | 0 (0) |
| Kidney |  |  |
| Clear cell carcinoma | 32 | 0 (0) |
| Papillary cell carcinoma | 17 | 0 (0) |
| Oncocytoma | 13 | 0 (0) |
| Lung cancer |  |  |
| Adenocarcinoma | 24 | 0 (0) |
| Bronchioloalveolar carcinoma | 21 | 0 (0) |
| Small cell carcinoma | 7 | 0 (0) |
| Lymhoepithelioma | 2 | 0 (0) |
| Melanoma | 11 | 0 (0) |
| Neuroblastoma | 3 | 0 (0) |
| Ovary, adenocarcinoma | 16 | 0 (0) |
| Pancreas, adenocarcinoma | 8 | 0 (0) |
| Prostate, adenocarcinoma | 3 | 0 (0) |
| Testicular cancer |  |  |
| Embryonal carcinoma | 5 | 0 (0) |
| Seminoma | 8 | 0 (0) |
| Teratocarcinoma | 13 | 0 (0) |
| Urinary bladder, transitional cell carcinoma | 11 | 0 (0) |
| **Total** | **598** | **37 (6)** |
